# Modulation of H-Reflex Depression with Paired-Pulse Stimulation in Healthy Active Humans

**DOI:** 10.1155/2017/5107097

**Published:** 2017-10-31

**Authors:** Preeti D. Oza, Shauna Dudley-Javoroski, Richard K. Shields

**Affiliations:** ^1^Department of Physical Therapy, University of the Pacific, 3601 Pacific Avenue, Stockton, CA 95211, USA; ^2^Department of Physical Therapy Rehabilitation Science, Roy J. and Lucille A. Carver College of Medicine, The University of Iowa, 500 Newton Road, 1-252 MEB, Iowa City, IA 52242, USA

## Abstract

Depression of the Hoffman reflex (H-reflex) is used to examine spinal control mechanisms during exercise, fatigue, and vibration and in response to training. H-reflex depression protocols frequently use trains of stimuli; this is time-consuming and prevents instantaneous assessment of motor neuronal excitability. The purpose of this study was to determine if paired-pulse H-reflex depression is reproducible and whether paired-pulse stimulation adequately estimates the depression induced by the more traditional ten-pulse train. H-reflexes were elicited via ten-pulse trains at 0.1, 0.2, 1, 2, and 5 Hz in ten neurologically intact individuals on two separate days. We measured the depression elicited by the second pulse (H2) and the mean depression elicited by pulses 2–10 (Hmean). H2 was consistent at all frequencies on both days (*r*^2^ = 0.97, *p* < 0.05, and ICC_(3,1)_ = 0.81). H2 did not differ from Hmean (*p* > 0.05). The results indicate that paired-pulse H-reflex depression has high between-day reliability and yields depression estimates that are comparable to those obtained via ten-pulse trains. Paired-pulse H-reflex depression may be especially useful for studies that require rapid assessment of motor neuronal excitability, such as during exercise, fatigue, and vibration, or to establish recovery curves following inhibition.

## 1. Introduction

Depression of the Hoffman reflex (H-reflex) is used to examine alpha motor neuronal presynaptic influences during exercise, vibration, fatigue, and recovery in response to either a short term or long term intervention [1–4]. The magnitude of H-reflex depression depends on the rate of stimulation and is greater at higher frequencies [5, 6]. H-reflex depression protocols use trains of repetitive stimulus pulses, usually at least 10 [5, 7]. At lower stimulation frequencies (e.g., 0.1 Hz), the experiment time may be considerable. This is disadvantageous for protocols involving exercise and/or fatigue, in which instantaneous assessment of spinal motor neuron excitability is necessary. H-reflex depression test time can be reduced via a paired-pulse technique which compares the magnitudes of only two reflex responses elicited at various frequencies [8–11].

Only one previous study investigated the between-day reliability of H-reflex depression elicited via paired-pulse stimulation [12]. The interpulse interval (IPI) used in this study (80 ms) was sufficiently short that the second H-reflex of the pair was recorded from a partially contracted muscle. Depression recorded after the second pulse may have reflected mechanical/architectural changes caused by muscle shortening beneath the EMG electrode [13]. For this reason, paired-pulse protocols with such a short IPI may be unsuitable for slow-twitch muscles like the soleus or for fatigued muscles exhibiting contractile slowing.

In the present study, we investigated paired-pulse H-reflex depression at a range of lower frequencies that avoid possible EMG artifacts due to contractile summation. The purpose of this study was to determine if paired-pulse H-reflex depression is consistent between days and whether paired-pulse stimulation adequately estimates the depression induced by the more traditional train of ten pulses.

## 2. Materials and Methods

### 2.1. Participants

Ten healthy young individuals [(mean ± SD) age = 27.4 ± 7.12 years, height = 1.73 ± 0.10 m, and weight = 70.5 ± 10.71 kg] were recruited for this study (5 males, 5 females). Participants signed an informed consent document approved by our institution's human subjects institutional review board.

Participants lay supine on a Kin-Com isokinetic dynamometer (Kin-Com 125 E Plus; Chattecx Corporation). The right hip and knee were flexed to 90° and the leg was supported with bolsters to prevent hip rotation during the experiment. The foot was secured to the footplate by Velcro straps and the subject's trunk was secured to the table with a belt. This position allowed clear access to the tibial nerve in the popliteal fossa. Participants were instructed to keep their leg muscles as relaxed as possible throughout the experiment.

### 2.2. Stimulation and Electromyographic Recordings

Soleus M-waves and H-reflexes were elicited by transcutaneous electrical stimulation of the tibial nerve. The tibial nerve was stimulated with a square pulse of 1000 *μ*sec delivered by a constant current stimulator (Model S88, Grass Medical, Quincy, MA, USA), with a current range of 50 *μ*A to 150 mA. The double-pronged surface stimulating electrode was positioned to yield an H-reflex response at the lowest stimulus intensity. The electrode was then secured to the popliteal fossa with an orthoplast splint and Velcro straps. The cathode was positioned proximal to the anode.

The skin surface over the soleus was abraded and scrubbed with alcohol. A bipolar silver-silver chloride surface electrode with 1 cm diameter and a fixed 2 cm interelectrode distance was placed over the soleus muscle at the junction of lower 1/3rd and upper 2/3rd of the lower leg, medial to the midline of the posterior calf, below the bulk of the gastrocnemius muscle. A reference electrode was positioned on the anterolateral surface of the leg. Soleus EMG signals (H-reflexes and M-waves) were on-site preamplified and then differentially amplified (Therapeutics Unlimited, Iowa City, IA, USA: 15 M*Ώ* at 100 Hz input impedance; 15–1000 Hz frequency response; 87 dB at 60 Hz common mode rejection ratio and gain of 500–10K times). EMG signals were displayed on an oscilloscope to verify the H-reflexes and the M-waves (Model TDS320, Tektronix, Beaverton, OR, USA). Analog signals were processed with a 16-bit analog-to-digital converter (Therapeutics Unlimited, Iowa City, IA, USA) at a sampling rate of 4000 Hz and then stored on a computer for later offline analysis.

### 2.3. Experimental Procedures

All subjects participated in two separate testing sessions separated by at least 7 days. Five maximum soleus M-waves (Mmax) were obtained by supramaximal electrical stimulation. Next, the stimulus intensity was reduced to elicit an H-reflex of about 25 ± 5% of Mmax amplitude. Ten H-reflexes at this stimulus intensity were recorded at 0.1 Hz, 0.2 Hz, 1 Hz, 2 Hz, and 5 Hz with a 15-second rest between the different frequencies. (An interval greater than 8 s is recommended to avoid the inhibitory effect of the preceding H-reflex [14].) The 0.1 Hz train was then repeated to verify that the electrode position had not changed.

### 2.4. Data Analysis

The peak-to-peak amplitudes of all H-reflexes and M-waves were determined in an automated fashion according to their latencies. The H-reflex integral was determined but not presented in this report as it revealed duplicate findings from the amplitude measurements. The H-reflex amplitudes were normalized to the Mmax for each subject. We examined four dependent variables: (1) the normalized amplitude of the first H-reflex (H1); (2) the normalized amplitude of the second H-reflex (H2) which illustrated H-reflex depression during paired-pulse stimulation; (3) the average of normalized amplitudes of the second to tenth H-reflexes (Hmean), which illustrated H-reflex depression during a 10-pulse stimulus train; and (4) the normalized amplitude of the small M-wave accompanying each H-reflex, which verified stimulus consistency.

### 2.5. Statistical Analysis

For day 1 H2 values, a one-way repeated measures analysis of variance (ANOVA) was used to determine whether H-reflex depression differed according to stimulation frequency. Multiple comparison tests (Tukey) were applied as indicated.

Three complementary approaches were used to assess the repeatability of H-reflex depression. The level of agreement of depression values between days was evaluated via a coefficient of correlation (*r*^2^) for H2 amplitude. A two-way repeated measures analysis of variance (ANOVA) was used to assess systematic differences in H2 amplitude between days across the different frequencies. Finally, for all frequencies of H2 combined, between-day reliability was evaluated via intraclass correlation coefficients (ICC_(3,1)_), together with standard error of measurement (SEM) and minimal detectable difference (MDD) [15].

In the context of human training studies, changes observed after an intervention or protocol must exceed the usual between-day variation in H-reflex depression. We calculated the percent difference between H2 values on the two experimental days to estimate typical between-day variation for future H-reflex depression studies.

Results of the above listed between-day tests indicated that H-reflex depression did not differ between days. This prompted us to pool day 1 and day 2 values during comparison of depression with paired pulses versus trains. We used a two-way repeated measures ANOVA (frequency × depression mode (paired pulse or train)) to compare the depression of the second pulse (H2) with the average depression within the train (Hmean).

To determine whether stimulus conditions remained uniform during the test protocol, we used a one-way repeated measures ANOVA to determine if H1 differed according to frequency or by day. Consistency of H1 responses across days prompted us to pool day 1 and day 2 data. We then used one-way repeated measure ANOVA to determine if the amplitude of the small M-waves preceding the H-responses varied according to frequency. We analyzed M1, M2, and Mmean in this manner.

The significance level for all tests was *p* < 0.05. Based on sample size calculations using a moderate effect size of 0.6, 10 subjects provided >80% power to detect a difference in H-reflex depression as computed by H2 versus Hmean.

## 3. Results

**Figure 1:**
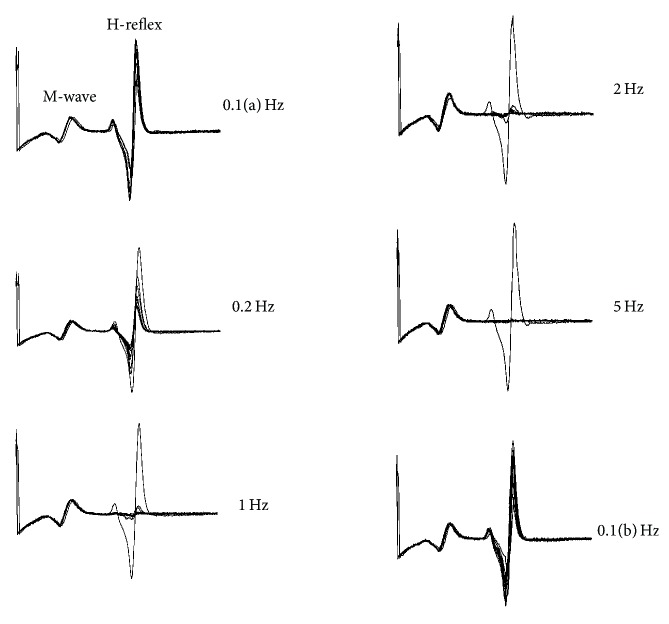
Representative example of 10-pulse H-reflex trains elicited at 0.1 Hz, 0.2 Hz, 1 Hz, 2 Hz, and 5 Hz. 0.1(a) Hz and 0.1(b) Hz were delivered at the start and end of the experimental protocol.

**Figure 2:**
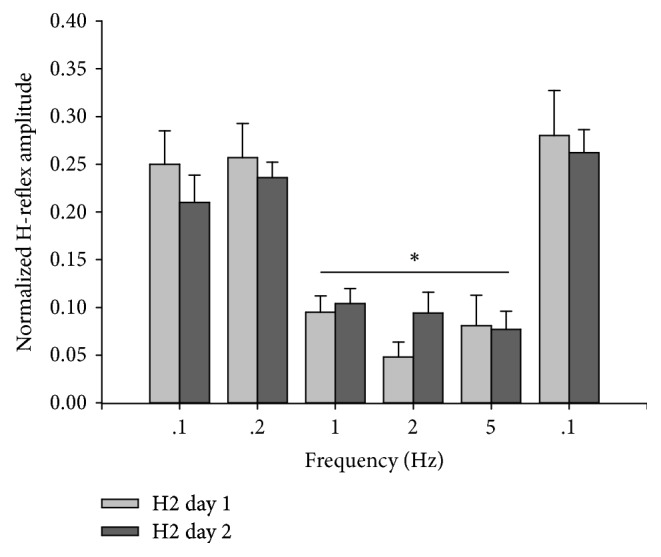
Normalized H2 amplitude at each frequency on the two experimental days. No difference in H2 existed between days at any frequency (all *p* > 0.05). *∗* = 1 Hz, 2 Hz, and 5 Hz significantly different from 0.1 Hz and 0.2 Hz (*p* < 0.05).

The magnitude of the H-reflex depression (H2) increased with frequency (Figure 1) (*p* < 0.05). Depression was significantly greater at 1, 2, and 5 Hz than at 0.1 and 0.2 Hz (all *p* < 0.003) (Figure 2). The magnitude of depression did not differ among 1, 2, and 5 Hz (all *p* > 0.82).

### 3.1. Reproducibility of the Depression

H2 responses between days were highly correlated (*r*^2^ = 0.97). No interaction existed between test day and stimulus frequency (*F*_(5,9)_ = 1.42,* p* = 0.23), indicating that the H-reflex behaved similarly between days at all frequencies. We therefore pooled frequencies to examine the concordance between day 1 and day 2 values. Between-day concordance via ICC_(3,1)_ was 0.81. A score ≥ 0.75 is a commonly used threshold for acceptable concordance [15]. Across all frequencies, the average percent difference in H2 between the two experimental days was 11.62% (42.25).

### 3.2. Paired-Pulse versus Train Depression

**Figure 3:**
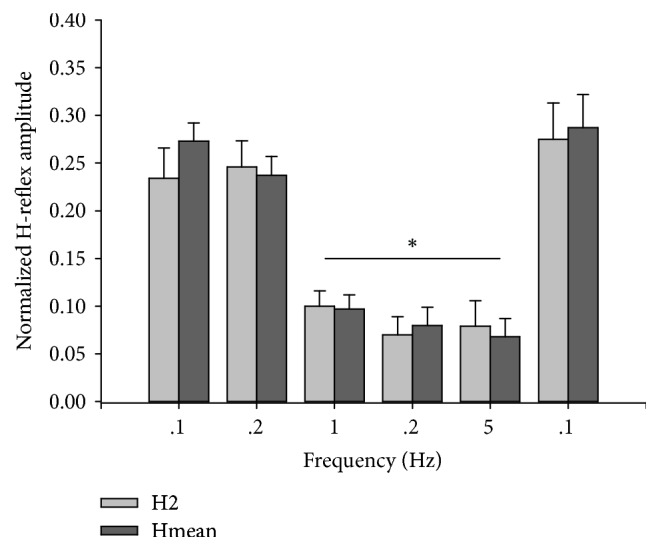
Normalized amplitude of H2 and the average of the H-reflex train (pulses 2–10, Hmean) at each frequency. No difference existed between H2 and Hmean at any frequency (all *p* > 0.05). *∗* = 1 Hz, 2 Hz, and 5 Hz significantly different from 0.1 Hz and 0.2 Hz (*p* < 0.05).

H2 and Hmean values were highly correlated (*r*^2^ = 0.98). No interaction existed between frequency and depression mode (H2 or Hmean) (*F*_(5,9)_ = 1.608,* p* = 0.177), indicating that H2 and Hmean behaved similarly to each other at all frequencies. There was no significant difference between the depression caused by the second pulse (H2) and the average depression of the train (Hmean) at any frequency (*F*_(5,9)_ = 0.720,* p* = 0.418) (Figure 3). Depression was significantly greater at 1, 2, and 5 Hz than at 0.1 and 0.2 Hz (all *p* < 0.001) and did not did not differ among 1, 2, and 5 Hz (all *p* > 0.860).

### 3.3. Stimulus Consistency

**Figure 4:**
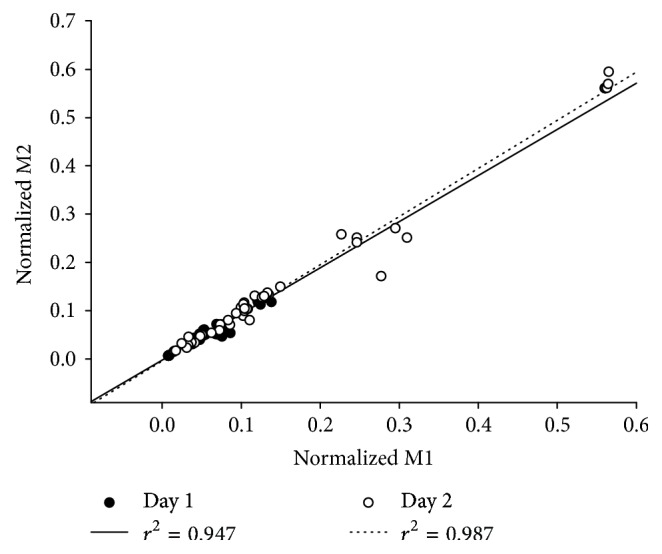
Correlation between M1 (the M-wave accompanying H1) and M2 (the M-wave accompanying H2) on the two experimental days for all subjects.

H1 did not differ significantly according to day or by frequency (*p* > 0.05). The average amplitude of the small M-wave on day 1 (M1) was systematically lower than the amplitude on day 2 (*p* < 0.05). However, M1 and M2 (small M-wave accompanying H2) were strongly correlated to each other on day 1 (*r*^2^ = 0.95) and on day 2 (*r*^2^ = 0.99) (Figure 4). The close correlation between the two M-waves indicates that the stimulus intensity was uniform for the first and the second H-reflexes on each particular day. It was therefore valid to calculate the paired-pulse H-reflex depression on both days. M1, M2, and Mmean (average of small M-waves 2–10) did not vary according to frequency (all *p* > 0.05). This analysis verified that the stimulation conditions remained uniform throughout the experiment.

## 4. Discussion

The major finding of this study was that H-reflex depression caused by the second pulse of a train was consistent with the average depression over the entire train. In experiments where the time constant of the change in H-reflex depression with repetitive stimulation is a variable of interest (e.g., studies of synaptic transmission), using a 10-pulse train continues to be a suitable methodologic approach. However, the results of the present study indicate that when the* magnitude* of depression is of interest, the second pulse is a reliable estimator of depression for the entire train. This paired-pulse depression showed less than 12% variation between experimental days, supporting its usefulness in electrophysiologic testing. Examination of paired-pulse depression may be particularly useful during fatigue protocols and vibration protocols and when examining the recovery curve after an intervention induces segmental inhibition. Importantly, the 2 Hz frequency was deemed the most optimal because it allowed the muscle to be fully relaxed, yielding less underlying stimulus artifact from shortening muscle architecture.

### 4.1. Reliability of Depression

The reliability of H-reflex amplitude (as a function of M-wave amplitude) has been previously examined. Maximal H-reflexes can be reliably elicited across days in multiple lower extremity muscle groups [16] in multiple joint positions [17] and at multiple levels of voluntary [17] and evoked contraction [18]. At low levels of recruitment (7–13% Mmax), H-reflex amplitude is reliable between days in subjects with incomplete spinal cord injury [19]. However, because the amplitude of the H-reflex appears to be modulated independently of H-reflex depression [9], the reliability of H-reflex depression between days must be separately examined.

The H-reflex depression observed in this study was consistent between days. This finding is noteworthy considering the possibility of day-to-day variability in neurologically intact subjects' biological systems. The reliability of paired-pulse depression at the low frequencies chosen for this study (ICC = 0.81) was comparable to the reliability previously reported for a higher frequency (12.5 Hz [12]). The reliability of paired-pulse depression is partially predicated upon between-day consistency of stimulation conditions. Although M1 was smaller on day 1 than on day 2, H1 responses did not differ between days. This indicates that, on the two test days, a uniform proportion of the soleus motor neuron pool was recruited during H-reflex testing. Because H-reflex depression varies as a function of the size of the conditioning stimulus (H1) [20], it was critical that H1 be as consistent as possible between test days.

### 4.2. Paired-Pulse Depression

Kohn and coauthors believed that the H-reflex depression caused by a train of 10 pulses is a graded response, suggesting an additive effect of depression with successive pulses [5]. However, in the present study we found no differences between H-reflex depression following the second pulse (H2) and the average depression for a train (Hmean). Due to the transient nature of fatigue, utilizing a shorter paired-pulse train would allow an examiner to more effectively capture the effects of fatigue. In addition, paired-pulse stimulation would likely induce less stimulation discomfort than multipulse trains. This feature could minimize nociception-induced fluctuations in descending drive to the motor neuronal pool. Uniform supraspinal input is a key factor regulating variability of H-reflex responses in neurologically intact subjects.

Earles and colleagues reported an ICC of 0.93 for paired H-reflex depression at an interpulse interval (IPI) of 80 ms [12], corresponding to a stimulus frequency of 12.5 Hz. The authors argued that, at this short IPI, descending supraspinal factors would not influence H2. While this may be valid, we believe that paired-pulse testing at short IPI's may not be methodologically advantageous for longitudinal training protocols. We noted more than 70% depression of H2 at the highest frequencies used in our study (2 Hz and 5 Hz). During pilot work, H2 depressed nearly 100% at 10 Hz. Thus if used in the context of a training study, 10 Hz stimulation could not reveal training-induced enhancements in H-reflex depression. Lower stimulating frequencies that yield only partial depression would allow detection of the full range of possible training-based responses. For this reason, we eliminated 10 Hz from the methodology of the present study and would recommend against using short IPIs for studies examining training-related changes.

### 4.3. Methodologic Strategies

As seen in Figure 2, depression occurred at 1, 2, and 5 Hz but not at 0.1 and 0.2 Hz. Multiple comparison procedures detected no difference in the magnitude of depression among 1, 2, and 5 Hz. Thus in addition to using paired-pulse stimulation to examine H-reflex depression, fatigue studies may also benefit from using just two paired-pulse frequencies: one that yields minimal depression (0.1 or 0.2 Hz) and one that yields substantial depression (1, 2 or 5 Hz). Compared to 0.1 Hz stimulation, 0.2 Hz stimulation would reduce the possible confounding effects of descending supraspinal input between H1 and H2. This, combined with shorter experiment duration, may support its use. As for the higher frequencies, 5 Hz stimulation would afford the least possibility of confounding supraspinal input between the paired pulses. However in slow muscles such as the soleus, summation of muscle contraction may begin at 5 Hz [21]. At 5 Hz, H2 may thus be evoked before the muscle relaxes completely from H1. Electromyographic signals arise from the volume of muscle lying directly under the recording electrode: altering this volume via contraction introduces variability to the EMG recording. Thus contractile summation should be avoided during H-reflex depression testing. While 2 Hz stimulation is subject to greater possible descending influence, it avoids the problem of contractile summation. We previously demonstrated the time course of recovery of H-reflex suppression after vibration [1]. A test (2 Hz) that minimally influences the state of spinal cord excitability may be viewed as ideal to determine the influence of interventions like exercise, fatigue, and mechanical stimulation.

## 5. Conclusions

H-reflex depression of the second pulse in a train was representative of the depression caused by the entire ten-pulse train. This paired-pulse depression was reliable between days, with less than 12% between-day variation. Paired-pulse assessment of H-reflex depression offers methodological advantages for assessing spinal control mechanisms during various interventions designed to regulate and adapt spinal cord excitability.
